# Hemophilic arthropathy in a patient with multi-joint replacement: A case report and literature review

**DOI:** 10.1097/MD.0000000000011163

**Published:** 2018-07-20

**Authors:** Wei Zhu, Xuxia He, Zenan Xia, Jiliang Zhai, Xisheng Weng

**Affiliations:** a Department of Orthopedics, Peking Union Medical College Hospital; b Department of Clinical Medicine, Chinese Academy of Medical Sciences and Peking Union Medical College, Tsinghua University, Beijing, China.

**Keywords:** hemophilia A, hemophilic arthropathy, multi-joint arthroplasty

## Abstract

**Rationale:**

Hemophilic arthropathy (HA) is a crucial morbidity and a major cause of joint pain and disability in patients with hemophilia A. Surgical methods, such as total joint arthroplasty, are of vital importance for end-stage HA treatment, but the feasibility and effects of multi-joint replacement surgery remain debatable.

**Patient concerns:**

A 24-year-old patient with advanced HA presented multiple joint pain. Physical examination revealed joint tenderness, swelling, and limited activity. Radiographs revealed bilateral knee joints and left elbow joint damage with joint space narrowing, articular facet erosion, and bone deformation.

**Diagnoses:**

The patient was diagnosed with hemophilic arthropathy with multi-joint lesions.

**Interventions:**

The key points of this case include arthropathy in multiple joints and the management of simultaneous total multi-joint arthroplasty. We performed bilateral total knee arthroplasty and total left elbow arthroplasty simultaneously after adequate preparations. Special attention was paid to factor VIII infusion, hemorrhage control, and other safety precautions perioperatively.

**Outcomes:**

After the surgery, no complications, such as infection or aseptic loosening, occurred, and the joints functioned well at follow-up.

**Lessons:**

The surgical outcome and safety of multi-joint replacement for HA are attested. Simultaneous multi-joint arthroplasty can ameliorate the quality of life for patients with hemophilia A.

## 1 Introduction

Hemophilia A is a congenital, X-linked recessive disorder characterized by deficiency of factor VIII and bleeding tendency.^[1]^ The most significant and disabling morbidity of hemophilia A is hemophilic arthropathy (HA), which is caused by repeated intra-articular bleeding. Feasible surgical methods include synovectomy, ankle debridement, arthrodesis, and most importantly, total joint arthroplasty.^[2]^ In general, satisfactory outcomes of total joint arthroplasty are reported in the literature, but problems such as infection, aseptic loosening, and arthrofibrosis have been noted during the complete disease course.^[3]^ A long-term rehabilitation program postoperatively and factor replacement therapy should be planned to realize utmost curative effect and reduce possible complications. Common joints damaged by hemophilia A include knees, ankles, elbows, shoulders, and hip joints. However, patients with HA with multi-joint arthroplasty are rarely reported, and relevant data are quite limited due to various medical and economic reasons. Here, we report a patient with HA with multiple affected joints and discuss current studies and feasible management in this circumstance.

## 2 Case report

A 24-year-old male with HA was admitted to our department with pain in multiple joints on May 23, 2011. The patient had a medical history of hemophilia A since the age of 3 and was intermittently treated with factor VIII. During these years, he sequentially developed left knee, left elbow, left hip, and right knee joint pain and swelling with limited activity and was soon diagnosed as HA. Initially, the joint manifestations could be largely relieved by factor VIII replacement therapy. Factor VIII inhibitor screening remained negative. Later, factor replacement therapy failed to achieve satisfactory effects, so in 2002 and 2006, he received left elbow synovectomy and left total hip arthroplasty, separately. In the subsequent years, the patient still suffered from the recurrent episodes of left elbow and bilateral knee joints hemorrhage, pain, and swelling. In recent 2 years, the frequency of joint hemorrhage had increased to approximately 2 times a week and only slightly relieved after factor VIII replacement therapy. Currently, the activity of those joints was limited to various degrees. Other medical history involved 2 cerebral hemorrhages 18 and 15 years ago, separately.

On physical examination, significant tenderness was noted in the left elbow joint with limited pronation and decreased grip strength. The preoperative Mayo elbow performance score (MEPS)^[4]^ was 55 for the left elbow. Moreover, knee valgus (left 20° and right 15°) was noted, and hyperextension, hyperflexion, and positive grinding test results were noted in both knee joints with a swollen and warm right knee. The preoperative Hospital for Special Surgery (HSS) knee scores^[5]^ were 58 for the left knee and 65 for the right knee.

**Figure 1:**
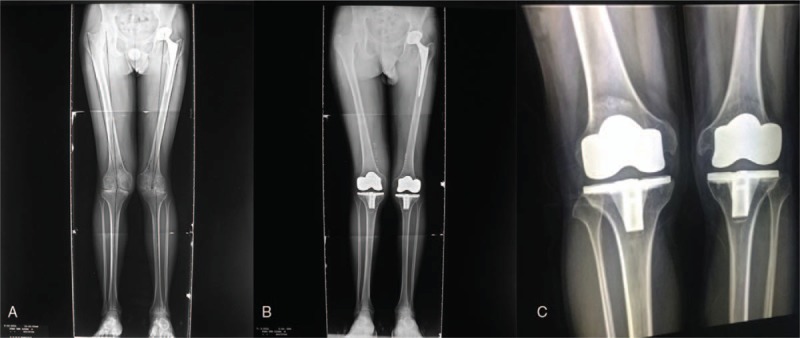
(A) Preoperative standing anterior radiograph of bilateral knee joint, revealing end-stage arthropathy with narrowing of the joint space and erosions of the articular facets. Note that the patient underwent total hip arthroplasty before these images were obtained. (B) Standing anterior radiograph of the same joints 3 months after bilateral total knee arthroplasty. (C) Standing anterior radiograph of the same joints 5 years after operation.

**Figure 2:**
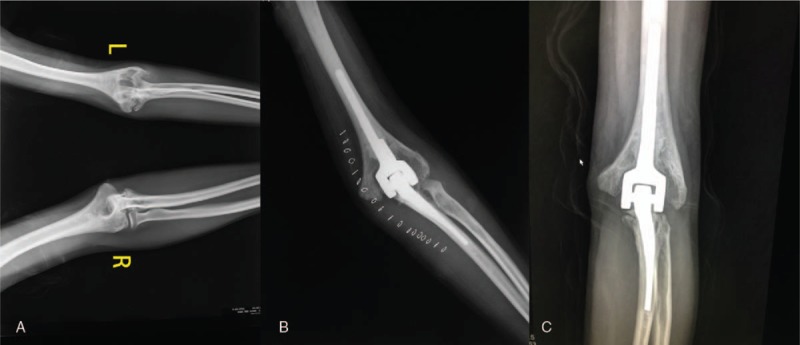
(A) Preoperative anterior radiograph of bilateral elbow joints, revealing end-stage arthropathy with narrowing of the joint space, erosions of the articular facets, and bone deformation of left elbow compared with the right elbow. (B) Anterior radiograph of the left elbow joint 3 months after left total elbow arthroplasty. (C) Anterior radiograph of the left elbow joint 5 years after the operation.

Bilateral knee joints and left elbow joint exhibit advanced arthropathy on radiographs (Figs. 1A and 2A). These joints present narrowing of joint space, erosions of the articular facets, and bone deformation to various degrees.

Our diagnosis was hemophilia A and HA of the left elbow joint, both knee joints, and left hip joint. The patient received left elbow synovectomy and left total hip arthroplasty, but the condition continued to deteriorate over time with worsening of the left elbow and both knee joints. Taking all of these factors into account, surgical methods were our top priority, and simultaneous total multi-joint replacement was indicated. Due to the complicated joint lesions and medical conditions, our preparations for this arthroplasty were far more sufficient than usual. Given that arthroplasty for patients with hemophilia A, particularly the simultaneous replacement of multiple joints, is challenging, the patient and his family were informed in detail of the possible benefits and risks of the surgery. We performed a full musculoskeletal assessment and thorough medical evaluation beforehand. Blood products were prepared for possible bleeding events. Then, our team performed bilateral total knee arthroplasty (Zimmer NexGen) and left total elbow arthroplasty (Zimmer) under tightly regulated factor VIII replacement therapy. Antibiotic prophylaxis was administered 30 minutes prior to surgery, and an additional dose was administered once during the operation. Local hemorrhage was carefully controlled to prevent secondary joint damage. Approximately 1800 mL blood was lost during the entire surgery. The patient received 900 mL blood by autotransfusion and 4 units of red blood cells plus 800 mL fresh frozen plasma by intraoperative infusion. During surgery, we observed hemarthrosis and villous synovial hypertrophy at the joints, and severe erosion of the articular surface and various degrees of bone deformation were noted. These findings confirmed the preoperative diagnosis and preoperative assessments. After surgery, hemostasis management, such as compressive bandage, factor VIII infusion, and rigorous monitoring of coagulation indicators, was performed. An early rehabilitation program was applied to achieve improved regain of function.

We managed factor VIII replacement therapy during perioperative period under the guidance of hematologists. On the day of surgery, 3000 U/12 h (the body weight of this patient is 63 kg) factor VIII (ADVATE) was administered intravenously followed by 2000 U/12 h on postoperative days 1 to 3 (POD 1–3). Then, on POD 4 to 6, a dose of 1500 U/12 h was administered followed by 1000 U/12 h over the following 6 weeks. Factor VIII inhibitor remained negative in perioperative tests.

At the follow-up, the patient's joints functioned well. The MEPS of the left elbow was 85, and the HSS score of knee joints were 71 (left) and 81 (right). On radiographs (3 months and 5 years after operation), the arthropathy of bilateral knee joints and left elbow joint was significantly relieved (Figs. 1B,C and 2B,C).

## 3 Discussion

Common manifestations of HA include joint pain, joint deformity, and soft tissue hematomas. Recurrent bleeding events result in synovial hyperplasia, joint erosion, and ultimately erosion of the underlying bone.^[6]^ Currently, numerous unresolved problems exist regarding the pathogenesis of HA.

The most commonly affected joints are knees, ankles, and elbows.^[7]^ Recurrent bleeding episodes within the same joint 4 times during 6 months can eventually lead to end-stage arthritis.^[8]^ The first joint involved in hemophilia is often the tibiotalar joint when the child starts to walk and gets hurt.^[9]^ Early diagnosis and early treatment before synovial damage and joint erosion develop are critical in the management of HA. The treatment for HA with multiple affected joints remains challenging at the current medical level. The primary goal is to relieve pain. In addition, restoring the range of motion and function of the joint represent additional goals. Although hemarthrosis aspiration, physical therapy and factor VIII replacement therapy can partly slow the progression of HA^[10]^ at early stage, surgical methods should be considered once the condition progresses to later period. Radiosynovectomy or arthroscopic synovectomy are recommended for early synovitis. Regarding end-stage incapacitated patients with severe joint tender and stiffness, surgery treatments, such as arthroscopic ankle debridement, arthrodesis, or arthroplasty, are last resorts.^[2]^ These methods are effective in relieving joint pain, correcting deformity, and restoring joint functions. To date, insufficient data have been reported to determine which surgical option is more preferable.^[11]^

In this case, the patient presented a sequence of severe damage in multiple joints, including the left elbow, left knee, right knee, and left hip, which complicated conditions. Research data on HA with lesions in multiple joints is lacking; thus, limited conclusions can be made to guide our management for this case. In fact, simultaneous multiple joint replacement surgery is still highly debated, and no consensus regarding its risks and benefits has been achieved. In terms of complications, data on comparison of single joint and multiple joint replacement and simultaneous and staged multi-joint replacement for patients with hemophilia A are lacking. However, one review^[12]^ analyzed data on bilateral total ankle arthroplasty (TKA) and observed an increased risk of complications with simultaneous bilateral TKA compared with unilateral TKA in patients without hemophilia. It was hypothesized that complications for simultaneous bilateral TKA in patients with hemophilia may include the more frequent use of factor VIII inhibitors and increased deep infection rate. These findings seem to imply that simultaneous multiple joint replacement for patients with HA would lead to higher risks compared with single joint replacement. On the contrary, Mortazavi et al^[13]^ compared the prognosis of 8 patients who underwent simultaneous bilateral TKA and 19 patients who underwent unilateral TKA and concluded that simultaneous bilateral TKA is a safe and cost-effective approach without augmenting the incidence of complications in patients with hemophilia. Thes et al^[14]^ conducted a retrospective case–control study and found that simultaneous bilateral TKA is associated with significantly lower costs of coagulation factors and longer hospital stay compared with staged bilateral TKA, but no significant difference in clinical outcome was noted. These data suggest that multi-joint replacement surgery for patients with hemophilia may be a good choice for end-stage multi-joint HA. However, to date, insufficient data are available to reach a definite conclusion.

Our case is characterized by manifestations of multiple joint lesions, including left elbow, left hip, and bilateral knees. The team performed simultaneous multiple joint replacement surgery with factor VIII supplement and hemostasis strategies. Regarding factor VIII replacement therapy, bolus injection and continuous infusion are 2 common methods. We chose continuous infusion, which seems to be more tolerable and effective than bolus injection in perioperative blood management.^[15]^

The operation and perioperative management effectively relieved joint pain and facilitated the regain of functions of these joints to a certain extent at the 3-month and 5-year follow-ups. Although simultaneous multi-joint arthroplasty improved the quality of life for this patient, further clinical research on the role of multiple joint replacement surgery in patients with hemophilia A is highly needed. The potential complications perioperatively must be taken into consideration.

## Author contributions

**Conceptualization:** Xisheng Weng.

**Data curation:** Xuxia He, Zenan Xia.

**Formal analysis:** Wei Zhu, Xuxia He.

**Investigation:** Wei Zhu, Xuxia He, Jiliang Zhai.

**Methodology:** Wei Zhu.

**Project administration:** Wei Zhu.

**Resources:** Zenan Xia.

**Supervision:** Wei Zhu, Xisheng Weng.

**Visualization:** Jiliang Zhai.

**Writing – original draft:** Xuxia He.

**Writing – review & editing:** Wei Zhu, Xuxia He.
